# Abuse of older adults before moving to old age homes in Pokhara Lekhnath Metropolitan City, Nepal: A cross-sectional study

**DOI:** 10.1371/journal.pone.0250639

**Published:** 2021-05-07

**Authors:** Mira Adhikari Baral, Bhagwati Khatri Chhetri, Pramila Bhandari

**Affiliations:** 1 Department of Adult Health Nursing, Pokhara Nursing Campus, Pokhara, Nepal; 2 Department of Nursing, Gandaki Medical College, Pokhara, Nepal; 3 Department of Nursing, Pokhara Technical Health Multipurpose Institute, Pokhara, Nepal; Sciensano, BELGIUM

## Abstract

**Introduction:**

The number of older individuals relocating from their homes to old age homes is increasing in Nepal. This study was conducted to assess the reasons why older people chose to move to old age homes, the status and forms of abuse they experienced, and the risk factors associated with their abuse before moving to old age homes.

**Materials and methods:**

This study was a cross sectional study conducted among older adults currently residing in all the old age homes of Pokhara Lekhnath Metropolitan City. Complete enumeration of the respondents was done and data was collected consecutively, using a semi-structured interview schedule, from all older adults aged 60 years and above who had been living in the homes for at least a month. The total sample size was 109. The collected data was analyzed using descriptive statistics and binary logistic regression was used as an inferential statistics to determine the predictors of abuse.

**Results:**

A majority of the respondents (56.0%) came to old age homes on their own volition, 24.7% reported that they came to the homes because they were physically weak and they had no one to care for them at their residence, while 11% reported that they were forcefully sent by their caregivers. Out of total respondents, 60.6% reported that they experienced some form of abuse before they came to the old age home: most frequent was caregiver neglect (34.9%) and verbal abuse (34.9%), while few experienced financial abuse (2.8%). Women were at a higher risk of abuse than men (*p*<0.05, OR = 4.430, CI = 1.695–11.577) prior to their transfer to old age homes.

**Conclusions:**

A majority of the older adults who transferred to old age homes in Pokhara Lekhnath Metropolitan city had been earlier abused by their caregivers, mainly through neglect and verbal abuse, and women were at a higher risk for abuse than men.

## Introduction

Globally, the population of older adults is increasing faster than any other age group [1–3]. In Nepal in 2011, the population of older adults aged sixty years and above comprised 8.1% of the total population [4] and this population is expected to reach to around 11% by 2031 [5]. In many developed nations, the ageing population/older adults are defined as individuals aged 65 years and above, however, in Nepal, the Senior Citizens Act, 2063 has defined older adults/senior citizens as those individuals who are aged 60 years and above [6].

Ageing, an inevitable phenomenon, can lead to various changes in the bio-psychosocial dimensions of older adults. This is a period when individuals experience a decline in their physical and mental health, have increased morbidities and financial challenges, experience the loss of loved ones, have decreasing social roles and influence, and have an increasing dependency on others for care and support [7,8]. In Nepal, family is considered the primary support for older adults and the Senior Citizens Act of Nepal has obliged families to take care of older individuals [6]. However, various social shifts, including increased preference for nuclear families, migration of the younger generation to urban areas or foreign countries for employment, and poor consideration of cultural values and norms that placed great respect on older adults by the younger generation, impose challenges to family support and wellbeing of older adults, making them socially isolated and insecure at their place of living, and increase their risk of being abused [9,10].

The social support system has a great role in easing life and promoting the health and wellbeing of older adults. The government of Nepal provides an old age allowance to all individuals aged above 70 years [11], but it is commonly reported as being insufficient to meet their basic requirements [12]. In addition to this, there is mismatch between life expectancy [13], the presence of chronic diseases [14–18] and the age at which older adults receive their old age allowance [19]. The government of Nepal delivers free health services to senior citizens aged above 60 years who cannot afford treatment [20], and also provides free health insurance to adults above the age of 70 years [21]. However, there are many issues related to quality of care, such as poor access to health services for older adults residing in rural areas, and there is also mismatch between demand and supply of medication [22]. Further, legal acts against abuse are very weak and there is no system for the management of the cases of abused victims in Nepal. Thus, the social security, health support and legal systems of Nepal are weak for addressing the needs of older adults.

Expression of abuse is still considered social stigma that discourages older adults from expressing the abuse they experience. Studies show that the prevalence of abuse of older adults in community is as high as around 50% [23,24]. Abuse results in many adverse physical and psychological consequences: these include physical injuries and psychological problems, such as depression, anxiety and post-traumatic stress disorder, and can even cause death [25–27]. Anecdotes show that in Nepal there are some reported cases of abuse of older adults that even led to their death [28,29].

In such circumstances, old age homes become a means of great support and security for older adults. The Senior Citizens Act of Nepal has allowed helpless and incompetent senior citizens to be placed in old age homes. In the case where a family member is not in a position to keep the senior citizen, the act has also allowed the family member to keep them in any care center [6]. In Nepal, three types of old age homes are available: Public, Private and community-run old age homes. Although older adults have to pay to live in private old age homes, public and community-run old age homes provide free services. Older individuals from better-off families choose to stay at private homes while others live in public and community-run homes.

Pokhara Lekhnath Metropolitan City, Kaski district, Nepal, has been highly affected by urbanization and modernization. The number of old age homes and older individuals moving to old age homes are also rapidly increasing. However, the reasons of relocation of older adults to old age homes and the status of their abuse before moving to old age homes is a poorly studied topic in this region. A study conducted in 2010 in Kaski district showed that there were very few reported cases of older abuse [29]. However, no any study has been conducted in this region, and in particular this city, to understand the reasons for transfer of older adults to old age homes and their status of abuse before moving to old age homes. The present study aims to fill the gap in knowledge by finding out the reasons associated with the movement of older adults to old age homes, and the status and the factors associated with abuse of older adults prior to their transfer to old age homes.

## Materials and methods

### Study design and participants

This is a cross-sectional study carried out in all the old age homes of Pokhara Lekhnath Metropolitan City. There are six old age homes in various wards of Pokhara Lekhnath Metropolitan City: five community-run homes and one privately-run home.

**Fig 1:**
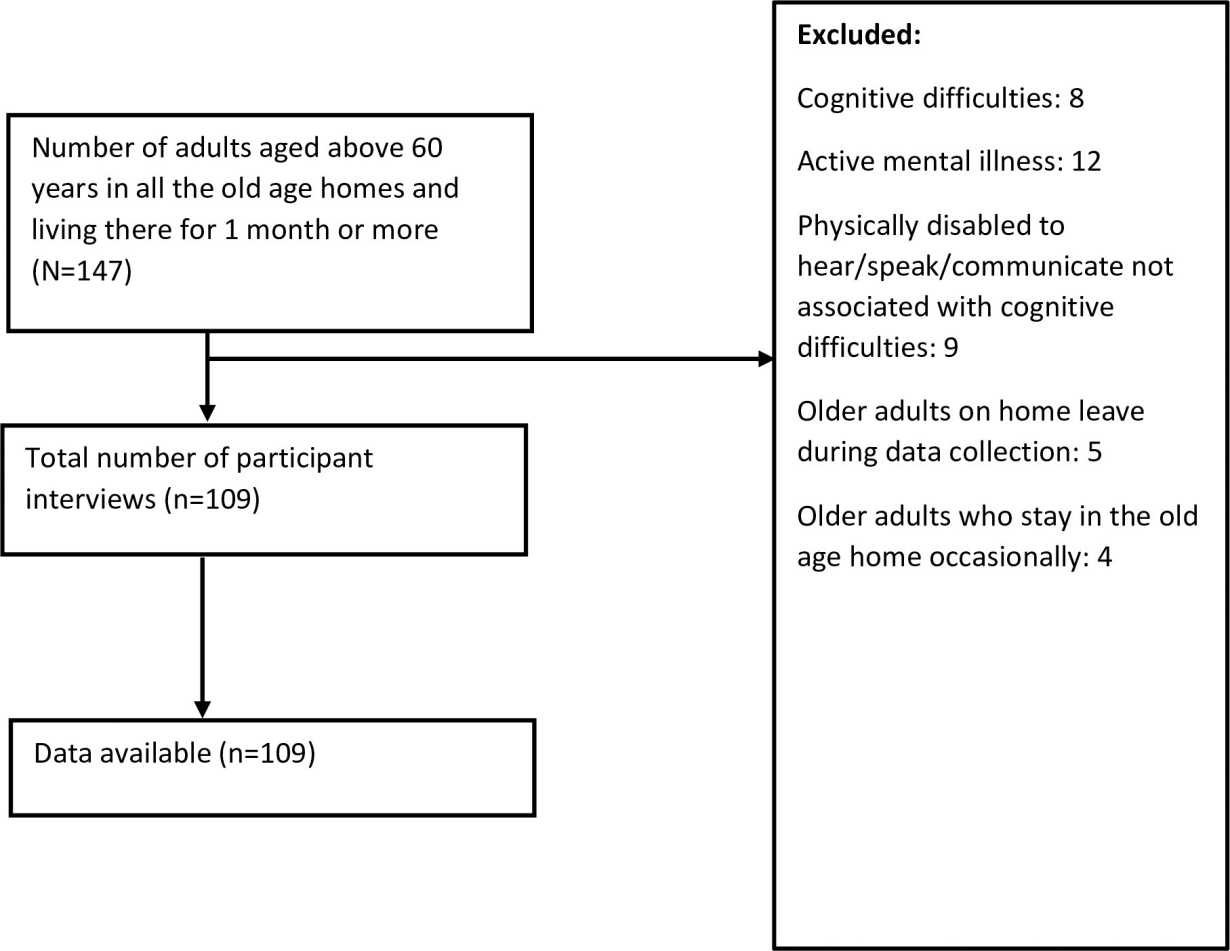
Selection of participants for the study.

The study population was older adults aged 60 years and above who had been living in the old age homes for a month or more. Those older adults who had been living in the old age home for less than one month, those who had hearing impairment and/or incomprehensible speech that impaired their ability to communicate, those who had cognitive difficulties and/or active mental illness, those who received care at old age homes intermittently, and those who were on home leave (absent) during the period of data collection were excluded from the study (Fig 1). Altogether, there were 147 adults aged 60 years and above who had been living in the respective old age homes for a month or more. However, eight older adults had cognitive difficulties, 12 had active mental illness, nine had physical disability that impaired their ability to communicate, four stayed at the old age home occasionally, and five were absent during the period of data collection and thus were excluded from the study. All of the respondents who met the inclusion criteria participated in the interview; the response rate was 100% (Fig 1).

### Data collection and study variables

#### Data collection tool

A semi-structured interview schedule was used to collect data from the respondents. This tool was developed by the researchers themselves being based on the key variables derived from an extensive literature review and several validated tools: the Elder Abuse Suspicion Index [30], the Elder Assessment Instrument [31], and the Katz index of independence on activities of daily living [32].This tool was developed because previously published tools were insufficient in depicting abuse in the Nepalese cultural setting. The questions were developed in a way to fit the social context of Nepal. The interview schedule was first prepared in English and then translated into the Nepalese language by the researcher, cross-checked by a bilingual expert, and then back-translated by a bilingual expert into English. This methodology was consistent and consistency was maintained with the Nepalese and English-language experts. Pre-testing of the tool was conducted in 15 older adults aged 60 years and above living in an old age home in Parbat, among those older adults who met the inclusion criteria, to check for clarity, adequacy, and consistency of the tool. The tool was checked for statistical reliability using Cronbach’s alpha; the value for the Chronbach’s alpha was 0.69, which is acceptable as reported in other studies [33,34].

#### Data collection procedure

The study was carried out after ethical clearance of the research proposal by the Institutional Review Committee of the Institute of Medicine at Tribhuvan University. The approved proposal along with a written request letter was submitted to the concerned old age homes of Pokhara Lekhnath Metropolitan City and formal permission was given. A meeting was then conducted with the board members in each of the old age homes: They were explained about the study, the nature of participation of the older adults in the study, the tool proposed for data collection, and the inclusion and exclusion criteria for the selection of participants. Upon our request, the old age homes provided us the demographic and medical information of patients. Then as per the records and information provided, the senior citizens who did not meet the inclusion criteria were excluded. Out of 147 respondents, 109 respondents participated in the study. Data was collected from August 2018 to December 2018. Prior to data collection, information was given to the respondents about the nature of the study and participants’ role in the research. The respondents were told that they had full authority to withdraw from the study without fear and explanation at any time during data collection, then informed written consent/thumb impressions was taken from the respondents. The average time required to complete the interview was 30–35 minutes. Data was collected by interviewing the older adults in a separate room in each of the old age homes to maintain privacy. Probing was done to prevent recall bias. Collected data was checked, and if something was found unclear, it was corrected then and there. Precaution was taken throughout the study to safeguard the rights and welfare of the respondents. Confidentiality was maintained throughout the study by omitting the name or any identifying information of the respondents.

#### Dependent variable

The primary outcome variable for this study was the abuse experienced by the older adults prior to moving to old age homes, being based on the self-report of the older adults. The answer choices were Yes/No. The abuse of the older adults in this study was assessed in six aspects: caregiver neglect, verbal abuse, physical abuse, sexual abuse, confinement, and financial abuse.

In this study, caregiver neglect refers to a failure or refusal of caregiver to provide food to the older individuals the same as to other family members or as per any medical condition, and a failure and refusal to provide clothes and treatment as needed. Verbal abuse in this study refers to speaking against the older adults in front of or besides others, scolding and expressing anger about nonsensical or minor matters and blaming them for things they did not do. Physical abuse refers to physical harm to the older adults caused by their caregivers through the act of intentionally pushing, hurting, and beating the elders. Sexual abuse refers to intentional touching on the private parts of older individuals against their will and forceful sexual contact with older adults against their will. Confinement refers to not allowing the older individuals to go to places or visit people they wanted. Financial abuse refers to the theft of money/assets, forcefully making the older adults sign papers related to the property/finance, and restricting them from spending their own money in areas they like to spend and/or spending the older adult’s money/property against their will. Positive responses on any of the above-mentioned elements were scored 1 and 0 for negative responses. The total number of questions that measured abuse was 13. Those older adults who scored 1 in any of the questions referring to the abuse was categorized as being abused before their arrival to old age homes.

#### Covariates

The following covariates were used in the study: age, sex, education status, marital status, place of residence, adequacy of annual income, living with status, chronic diseases and dependence on others for activities of daily living. In particular, education status refers to the illiteracy or literacy status (both formal and/or informal) of the respondents, marital status refers to being married or the status of being unmarried, divorced, separated or widow/widower. Adequacy of annual income refers to sufficiency of annual income for basic living expenses throughout the year. Living with status refers to living arrangement of older adult with their family members or with relatives, friends or living alone, chronic diseases refers to presence of chronic disorders among the older adults before their arrival into the old age homes and dependence of daily living activities of daily living refers to dependence of older adults on others for any of the following: bathing, brushing, clothing, eating, toileting and moving from one place to other.

#### Data analysis

Collected data was edited, organized, coded, and entered in SPSS version 16 for analysis. The data was analyzed using descriptive statistics such as frequencies, percentage, mean and standard deviation. Binary logistic regression was used to determine the factors associated with abuse of the respondents before their arrival into the old age homes. For bivariate analysis, each independent variables was included separately in the binary regression model. For multivariate analysis, the covariates age, sex, marital status, education status, place of residence, adequacy of annual income, living with status, chronic diseases and dependency on others for activities of daily living were included in binary logistic regression model. The level of significance was considered at *p*-value ≤0.05.

## Results

### Sample study characteristics

The findings of the study show that 53.2% of the older adults were from the age group 60 years to 70 years and 60.6% of the respondents were female. The age of participants ranged from 60 to 100 years, and the mean age of respondents was 71.70 years ± 7.67 standard deviation (SD). A majority of the respondents were illiterate (76.1%) and from rural area of residence (67%). Regarding marital status, 41.3% of the older adults were married and 36.7% were either a widow/widower, separated, or divorced. Among all the married, widowed, separated and divorced older adults, 54.10% had children. Among the women who were married/widowed/separated/divorced, 50.0% had children. A majority of the older adults (81.7%) reported farming as their major occupation before they went to live in the old age homes and 70.6% of the respondents reported that they had an annual income sufficient for their basic living expenses throughout the year before their transfer to the old age home. In regard to the decision to come to the old age home, 80.7% reported that it was their own decision to come to old age home. The proportion of respondents who reported living with their families before their arrival to old age homes was 45.9%. With respect to living in old age homes, a majority of the respondents (53.2%) reported that they had been living in the old age home for less than or equal to 5 years (Table 1).

**Table 1:** Background information of older adults residing in old age homes (n = 109).

Background characteristics	Frequency	Percentage
**Age** ^**#**^		
60 years-70 years	58	53.2
71 years and above	51	46.8
**Sex**		
Male	43	39.4
Female	66	60.6
**Education**		
Illiterate	83	76.1
Literate	26	23.9
**Place of residence**		
Village Development Committee (VDC)	73	67.0
Municipality	36	33.0
**Marital status**		
Married	45	41.3
Unmarried	24	22.0
Widow/widower/separated/divorced	40	36.7
**Married older individuals including widow/widower/married/divorced having children (n = 85)**	**46**	**54.1**
**Females Married/widow/separated/divorced having children (n = 58)**	**29**	**50.0**
**Occupation before coming to old age homes**		
Farming	89	81.7
Service	12	11.0
Others (daily wages, begging, mason, small business, priest)	8	7.3
**Adequacy of annual income throughout the year**	77	70.6
**Decision making to come to old age home**		
Self-decided	88	80.7
Decided by someone else	21	19.3
**Before coming to old age home, resided with**		
Family	50	45.9
Alone	32	29.3
Other relatives	22	20.2
Friends	5	4.6
**Duration of stay at the old age home**		
0–5 years	58	53.2
6–10 years	36	33.0
11–15 years	10	9.2
>15 years	5	4.6

# Overall mean age: 71.70±7.67 years (minimum 60 years, maximum 100 years).

The health-related information of the older adults before coming to old age homes is represented in Table 2. Fifty five percent of the older adults reported that they had chronic disease conditions before they came to the old age home and among them only 50% reported that they got regular treatment of the disease conditions before their arrival to the old age home. However, after the older individuals transferred to old age homes, treatment rates for the chronic diseases increased to 81.67%. The major health problems among the respondents were heart disease and hypertension (21.1%) followed by gastrointestinal problems (11.9%). Before coming to old age homes, 6.4% of the respondents were dependent on others for basic activities of daily living, however, after coming to old age homes, 10.1% of the respondents depended on others for basic activities of daily living.

**Table 2:** Health-related information of older adults transferred to the old age homes (n = 109).

Health-related characteristics before coming to the old age home	Frequency	Percent
**Reported chronic disease conditions present before coming to the old age home (n = 109)**	60	55.0
**Got regular treatment for chronic diseases in the past (n = 60)**	30	50.0
**Getting regular treatment for chronic diseases at present (n = 60)**	49	81.6
**Diseases among older adults before residing in old age homes (n = 109)**		
Heart and hypertension	23	21.1
Gastro-intestinal problems (Chronic gastritis, chronic indigestion)	13	11.9
COPD	9	7.3
Arthritis	6	5.5
Mental problems (depression, anxiety, PTSD)	5	4.6
Diabetes	4	2.8
Others (stroke, leprosy)	2	1.8
**Dependence on others for any of the basic activities of daily living (n = 109)**		
Before coming to the institution	7	6.4
After coming to the institution	11	10.1

### Major reasons for deciding to move to old age homes

Table 3 shows that majority of the respondents (56.0%) came to the old age home on their own wish while being able to care for themselves, 24.7% reported that they came to old age home because of being unable to care for themselves and having no one to care for them, whereas 11% reported that they were forced to move into the old age home.

**Table 3:** Major reasons why older adults chose to move to old age homes (n = 109).

Reasons	Frequency	Percent
One’s own wish while being able to care for oneself	61	56.0
Having no one to care and not being able to care for own self	27	24.7
To fulfill children’s/caregiver’s wish	9	8.3
Forcefully sent by caregivers	12	11.0

### Abuse experienced by the older adults prior to their transfer to old age homes

Various forms of abuse experienced by the older adults before coming to the old age homes are shown in Table 4. A majority of the respondents (33%) reported that they had no access to medical treatment as needed before coming to old age homes followed by 27.5% of the respondents who reported that they were scolded by their caregivers about nonsensical/minor things, while few of them (0.9%) reported that they were forced to sign papers related to their property by their caregivers.

**Table 4:** Areas of abuse of older adults before coming to old age homes (n = 109).

Characteristics indicating abuse before coming to old age home	Frequency	Percentage
**Caregiver neglect**		
No access to food as other members of family	20	18.3
No access to clothes as other members of family	23	21.1
No access to medical treatment	36	33.0
**Confinement**		
Restrictions on visiting people or places	10	9.2
**Verbal abuse**		
Speak against them in a humiliating/threatening manner	26	23.8
Scold/express anger over nonsensical/minor issues	30	27.5
Blame for things they did not do	19	17.4
**Physical/sexual abuse**		
Anyone beat/harmed physically	6	5.5
Touched private body parts against their will	8	7.3
Try to establish/established forceful sexual relations	8	7.3
**Financial**		
Theft of money/property	2	1.83
Restriction on/forced to spending own money against their will	3	2.8
Forced to sign property papers	1	0.9

This study revealed that 60.6% of the older adults experienced some form of abuse before they moved to the old age home. The mean score for abuse was 1.76±2.15 SD. The minimum and maximum score for abuse (among those abused) was 1 and 9, respectively. Among the abused older adults, a majority of them reported that they experienced caregiver neglect (34.9%) and verbal abuse (34.9%), and 9.2% reported confinement, followed by sexual abuse (7.3%), physical abuse (5.5%) and financial abuse (2.8%) (Table 5).

**Table 5:** Forms of abuse experienced by older adults before coming to old age home (n = 109).

Forms of abuse experienced by older adults	Frequency	Percentage
**Abuse total** **#**	**66**	**60.6**
Physical abuse	6	5.5
Caregiver neglect	38	34.9
Verbal abuse	38	34.9
Confinement	10	9.2
Financial abuse	3	2.8
Sexual abuse	8	7.3

#: Mean score (1.76±2.15) (among abused, minimum score for abuse 1, maximum score 9).

### Factors associated with abuse of the older adults prior to transfer to old age homes

After adjusting for age, sex, education, marital status, place of residence, adequacy of annual income, living with status, chronic disease, and dependency status, this study showed that women were at an increased risk for being abused than men (p = <0.05, AOR = 4.430, CI = 1.695–11.577) prior to their transfer into old age homes (Table 6).

**Table 6:** Factors associated with abuse of older adults transferred to old age homes (n = 109).

Background characteristics	Crude Odds(95% CI)	Adjusted Odds(95%CI) ^¤^
**Age**		
Up to 70 years	2.508(1.141–5.516)^a^	2.318(0.951–5.650)
71 years and above	1	1
**Sex**		
Female	3.125(1.399–6.981)^b^	4.430(1.695–11.577)^b^
Male	1	1
**Education**		
Illiterate	0.481(0.182–1.268)	0.318(0.090–1.128)
Literate	1	1
**Marital Status**		
Unmarried/widowed/separated/divorced	0.460(0.205–1.035)	0.964(0.343–2.711)
Married	1	1
**Place of Residence**		
Municipality	1.476(0.642–3.399)	1.062(0.403–2.802)
VDC	1	
**Adequacy of annual income for 1 year**		
Inadequate	0.933(0.402–2.163)	0.734(0.268–2.007)
Adequate	1	1
**Before coming to the old age home, lived with**		
Other than family	0.474(0.215–1.047)	0.643(0.242–1.712)
With family	1	1
**Before coming to old age home**		
Did not have chronic disease	0.483(0.221–1.054)	0.539(0.224–1.297)
Had chronic disease	1	1
**Before coming to old age home**		
Independent for ADLS	12.154(0.457–10.141)	1.975(0.326–11.984)
Dependent on others for ADLS	1	1

^a^ = p<0.05.

^b^ = p<0.01.

^¤^: The variables included in adjustment model were age, sex, education, marital status, place of residence, adequacy of annual income, living with status, chronic disease, and dependency for ADLs.

## Discussion

Development, modernization and urbanization have brought changes in various aspects of Nepalese society. Recently, the number of old age homes have been increasing in Nepal. In the past, the religious places like temples were used to give shelter to the homeless, however, with time, the concept of old age home is changing. A majority of the old age homes in Nepal are established to provide basic services like food and shelter to the destitute free of cost while there are other age care homes that focus on providing advanced care facilities to older adults while also receiving fees for their services.

Older adults move to old age homes for several reasons. Our findings show that although 56% of the respondents came to the old age home of their own will despite being able to care for themselves, 24.8% reported that they had no one to care them during their declining health while others reported that they were directly or indirectly forced by others to move to the old age homes. This signals that lack of support is a major reason for movement of older individuals into old age homes in Nepal. Further, this study showed that 78% of the senior citizens were or had been married (including widow/widower/separated/divorced) and among them, only 54.1% of the respondents had children. Children are the primary careers of older adults in Nepal [6,9,10], and not having children may have caused lack of support to the older adults and motivated or forced them to move to the old age homes. The finding is similar to studies conducted in India, which showed that lack of support and poor physical and mental health were the reasons for elderly to move to old age [35,36]. The consistency of the result may be attributed to similarity in the setting. Further, most of the old age homes of Pokhara Lekhnath are community-run old age homes and they provide free lodging and food facilities to the older adults. This may have attracted the helpless older adults to come to old age homes to secure their living.

The findings of the current study show that although 55% of the respondents had some form of chronic disease before they came to the old age home, only 50% of them were getting regular treatment. This indicates that the older adults are suffering with disease symptoms in the community. Anecdotes suggest that Nepalese often develop chronic disease at a relatively early age, in their late fifties and early sixties [37]. However, they are eligible for the free health insurance provided by the government once they complete 70 years [21]. Further, research shows that there is poor accessibility of the service and a relatively high cost of transportation to the referral services, and there is a poor supply of all drugs via the scheme [22]. All these factors could have hindered the unwell older adults from receiving treatment for their health conditions. After their transfer to old age homes, 81.6% of the respondents who had chronic diseases reported that they were getting regular treatment/medications for their diseases. Many of the community-run old age homes depend on volunteer medical treatment from many non-governmental organizations (NGOs) and other organizations. In some cases, expensive drugs and treatment may not be available and, some older adults do not get the treatment they need. Furthermore, this study showed that the mean age of the respondents living in old age homes was 71.7 years, which indicates that a majority of the older adults fell below 70 years of age and were not eligible to receive the free health insurance for their treatment. Hence, this situation should be considered seriously by policymakers and implementers, and policy should be reformed to include free treatment provisions for older citizens at an earlier age, and health services should be made feasible and accessible at every level of health care.

This study showed that 60.6% of the older individuals experienced some form of abuse before they transferred to the old age home. The result is similar to another study conducted in the old age homes of Kathmandu, which showed that 57.9% of the older adults experienced some form of abuse before coming to the old age homes [38]. The studies conducted in community settings of Nepal showed a relatively lower prevalence of abuse of the senior citizens [24,39,40]. The finding is also higher than studies conducted in communities in India [41,42], China [43], and the USA [44,45]^.^ This difference might be related to the independence that the older adults have in old age homes to freely talk about abuse they experienced compared to the community setting. Further, since there are many community-run old age homes that aim to support the most deprived/affected older population of the community in Pokhara Lekhnath Metropolitan City, this may have contributed to a high prevalence of abuse among the older adults residing in old age homes. The higher reporting of abuse by the older adults signal that being abused is a push factor for residents moving to the old age homes of Pokhara Lekhnath Metropolitan City. The highest reported forms of abuse were caregiver neglect (34.9%) and verbal abuse (34.9%), which are frequently highly reported in studies elsewhere [23,38,43–45].

Talking about sex is a social taboo in Nepal. Despite this, 7.3% of the respondents reported that they experienced sexual abuse and all were female. The finding is significantly higher than the finding reported by other studies conducted in community settings [23,24,40,44]. This reflects that some women who come to old age homes were also the victims of sexual abuse. This may be related to poor legal provisions against sexual abuse and poor security of the older females caused by social isolation due to family disintegration and dominance of males over females in Nepal. This is a serious concern that needs to be addressed in the community at the local level by initiating awareness and women empowerment programs and conducting training for women in the community to equip them with self-defense skills so that they can protect themselves from being abused. Further, the legislative acts related to abuse should be reformed to include strict punishment to the abuser so that people become discouraged to sexually abuse others. In addition, a formal system for reporting the abuse, and protecting and treating the abused should be established for managing the victims of abuse.

This study showed that after adjusting the variables age, sex, education, marital status, place of residence, adequacy of annual income, living with status, presence of chronic disease, and dependence on others for activities of daily living, women had a higher risk for abuse than men (p = <0.05, OR = 4.430, CI = 1.695–11.577). This finding is consistent with another study conducted in Nepal [23,46], Thailand [47] and the USA [45], which showed that older women are at a higher risk for abuse than men. This may be related to financial dependency of older females over males, erosion of social values and norms that provided older women with respect from children and in-laws in the family, and social isolation caused by increased preferences for nuclear family in Nepalese society [9,10]. However, the finding of this study is contrary to the findings from a study in Negeri Sembilan state, Malaysia, and Nepal which showed that men were at a higher risk for abuse than women [48,49]. The inconsistency of the result may be related to variances in operational definition of abuse and study population.

This study showed that there is a high prevalence of caregiver abuse among the older adults who moved to old age homes. Although the government has made some provisions for the social security of older adults, it seems to be inadequate to support them. It is very necessary for the government to review the effectiveness of the health and social support programs for older individuals and revise them in order to ensure that most of the older adults benefit from those programs.

Further, there are no systems and legal provisions for reporting and punishing the abusers unless the death of an abused older adult occurs, and no system has been formed to manage and treat the abused older adult. It is very necessary for the government to develop a formal mechanism for reporting abuse and care for the abused older adults at a local and national level. It is very necessary to reform and strengthen laws and acts against the abusers. Further, the local and national governments should design and implement integrated community programs on the prevention of abuse at various levels of the community.

This study is limited to the old age homes of Pokhara Lekhnath Metropolitan City and thus cannot be generalized to other settings. Although probing was done and since this study was focused on previous experiences, there is a chance of recall bias. Moreover, our study represents the self-reported abuse based on the perception of the older adults and not on the findings of direct observation and validation from caregivers and thus may differ with the perception of the older adults. Further, since this study excludes older individuals with cognitive difficulties, active mental illness, and physical illness that impair their ability to communicate, the findings may be insufficient to represent the real picture of all the older individuals living in the old age homes of the metropolitan city.

## Conclusions

This study concluded that the majority of the older adults residing in old age homes of Pokhara Lekhnath Metropolitan City came to old age homes by their own will, without the coercion of others, but a majority of them were the victims of abuse from their caregivers before their arrival into the old age homes. A majority of the older adults experienced caregiver neglect and verbal abuse, while few experienced financial abuse. Older women were at a higher risk for abuse than men.

Expression about is considered a social stigma in Nepal, and therefore is a common hidden problem in Nepalese society and age homes are a means of support for the older adults who are abused in their families and communities. It is recommended that the government conduct a large and an in-depth study into the area of abuse of older adults living in community and institutional settings at various levels and use the findings as a basis to develop and implement necessary policies to strengthen the social security, health and wellbeing of the older population and ensure that their rights are protected, that decrease their vulnerability to being abused.

Further, the local and national governments should develop integrated programs on prevention and management of abuse of older adults at various levels. Awareness programs should be conducted at the community level to educate community people about the rights of older adults and their responsibilities towards caring the older adults as imposed by the law. Further, the legal penalties for abuse should be made stricter, and a system should be developed for reporting abuse and management/treatment of victims. This may reduce the risk of abuse of older adults and the consequences of abuse.

## Supporting information

S1 Fig Selection of participants for the study. dx.doi.org/10.17504/protocols.io.bpcimiue.
(PDF)

S1 Table Background information of older adults residing in old age homes. (PDF)

S2 Table Health related information of older adults transferred to the old age homes. (PDF)

S3 Table Major reasons why older adults chose to move to old age homes. (PDF)

S4 Table Areas of abuse of older adults before coming to old age homes. (PDF)

S5 Table Forms of abuse experienced by older adults before coming to old age home. (PDF)

S6 Table Factors associated with abuse of older adults transferred to old age homes. (PDF)
