# Supplementary figures and images for: Abuse of older adults before moving to old age homes in Pokhara Lekhnath Metropolitan City, Nepal: A cross-sectional study
Source: PLoS One. 2021 May 7;16(5):e0250639. doi: 10.1371/journal.pone.0250639 (PMC8104417; doi:10.1371/journal.pone.0250639)

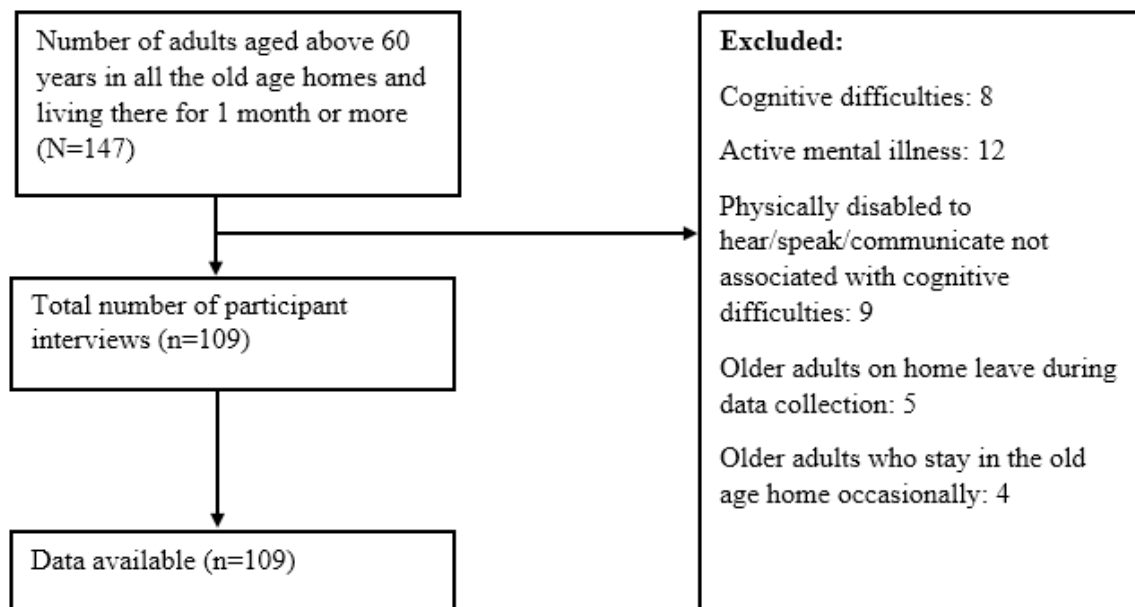

**Figure 1: Selection of participants for the study**

Supplement: S1 Fig — dx.doi.org/10.17504/protocols.io.bpcimiue. (PDF) [file pone.0250639.s001.pdf]
