# Supplementary material for: Abuse of older adults before moving to old age homes in Pokhara Lekhnath Metropolitan City, Nepal: A cross-sectional study
Source: PLoS One. 2021 May 7;16(5):e0250639. doi: 10.1371/journal.pone.0250639 (PMC8104417; doi:10.1371/journal.pone.0250639)
Supplement: S1 Table — (PDF) [file pone.0250639.s002.pdf]

Table 1: Background information of older adults residing in old age homes (n=109)

**Background characteristics**	**Frequency**	**Percentage**
**Age #**		
60 years-70 years	58	53.2
71 years and above	51	46.8
**Sex**		
Male	43	39.4
Female	66	60.6
**Education**		
Illiterate	83	76.1
Literate	26	23.9
**Place of residence**		
Village Development Committee (VDC)	73	67.0
Municipality	36	33.0
**Marital status**		
Married	45	41.3
Unmarried	24	22.0
Widow/widower/separated/divorced	40	36.7
**Married older individuals including widow/widower/married/divorced having children (n=85)**	**46**	**54.1**
**Females Married/widow/separated/divorced having children (n=58)**	**29**	**50.0**
**Occupation before coming to old age homes**		
Farming	89	81.7
Service	12	11.0
Others (daily wages, begging, mason, small business, priest)	8	7.3
**Adequacy of annual income throughout the year**	**77**	**70.6**
**Decision making to come to old age home**		
Self-decided	88	80.7
Decided by someone else	21	19.3
**Before coming to old age home, resided with**		
Family	50	45.9
Alone	32	29.3
Other relatives	22	20.2
Friends	5	4.6
**Duration of stay at the old age home**		
0-5 years	58	53.2
6-10 years	36	33.0
11-15 years	10	9.2
>15 years	5	4.6

# Overall mean age: 71.70±7.67 years (minimum 60 years, maximum 100 years)
