# Supplementary material for: Abuse of older adults before moving to old age homes in Pokhara Lekhnath Metropolitan City, Nepal: A cross-sectional study
Source: PLoS One. 2021 May 7;16(5):e0250639. doi: 10.1371/journal.pone.0250639 (PMC8104417; doi:10.1371/journal.pone.0250639)
Supplement: S2 Table — (PDF) [file pone.0250639.s003.pdf]

Table 2: Health-related information of older adults transferred to the old age home (n=109)

**Health-related characteristics before coming to the old age home**	**Frequency**	**Percent**
**Reported chronic disease conditions present before coming to the old age home (n=109)**	**60**	**55.0**
**Got regular treatment for chronic diseases in the past (n=60)**	**30**	**50.0**
**Getting regular treatment for chronic diseases at present (n=60)**	**49**	**81.6**
**Diseases among older adults before residing in old age homes (n=109)**		
Heart and hypertension	23	21.1
Gastro-intestinal problems (Chronic gastritis, chronic indigestion)	13	11.9
COPD	9	7.3
Arthritis	6	5.5
Mental problems (depression, anxiety, PTSD)	5	4.6
Diabetes	4	2.8
Others (stroke, leprosy)	2	1.8
**Dependence on others for any of the basic activities of daily living (n=109)**		
Before coming to the institution	7	6.4
After coming to the institution	11	10.1
