# Supplementary material for: Abuse of older adults before moving to old age homes in Pokhara Lekhnath Metropolitan City, Nepal: A cross-sectional study
Source: PLoS One. 2021 May 7;16(5):e0250639. doi: 10.1371/journal.pone.0250639 (PMC8104417; doi:10.1371/journal.pone.0250639)
Supplement: S3 Table — (PDF) [file pone.0250639.s004.pdf]

Table 3: Major reasons why older adults chose to move to old age homes (n=109)

Reasons	Frequency	Percent
One's own wish while being able to care for oneself	61	56.0
Having no one to care and not being able to care for own self	27	24.7
To fulfill children's/caregiver's wish	9	8.3
Forcefully sent by caregivers	12	11.0
