# Supplementary material for: Abuse of older adults before moving to old age homes in Pokhara Lekhnath Metropolitan City, Nepal: A cross-sectional study
Source: PLoS One. 2021 May 7;16(5):e0250639. doi: 10.1371/journal.pone.0250639 (PMC8104417; doi:10.1371/journal.pone.0250639)
Supplement: S4 Table — (PDF) [file pone.0250639.s005.pdf]

**Table 4: Areas of abuse of older adults before coming to old age homes (n=109)**

| Characteristics indicating abuse before coming to old age home | Frequency | Percentage |
|---|---|---|
| **Caregiver neglect** | | |
| No access to food as other members of family | 20 | 18.3 |
| No access to clothes as other members of family | 23 | 21.1 |
| No access to medical treatment | 36 | 33.0 |
| **Confinement** | | |
| Restrictions on visiting people or places | 10 | 9.2 |
| **Verbal abuse** | | |
| Speak against them in a humiliating/threatening manner | 26 | 23.8 |
| Scold/express anger over nonsensical/minor issues | 30 | 27.5 |
| Blame for things they did not do | 19 | 17.4 |
| **Physical/sexual abuse** | | |
| Anyone beat/harmed physically | 6 | 5.5 |
| Touched private body parts against their will | 8 | 7.3 |
| Try to establish/established forceful sexual relations | 8 | 7.3 |
| **Financial** | | |
| Theft of money/property | 2 | 1.83 |
| Restriction on/forced to spending own money against their will | 3 | 2.8 |
| Forced to sign property papers | 1 | 0.9 |
