# Supplementary material for: Abuse of older adults before moving to old age homes in Pokhara Lekhnath Metropolitan City, Nepal: A cross-sectional study
Source: PLoS One. 2021 May 7;16(5):e0250639. doi: 10.1371/journal.pone.0250639 (PMC8104417; doi:10.1371/journal.pone.0250639)
Supplement: S5 Table — (PDF) [file pone.0250639.s006.pdf]

Table 5: Forms of abuse experienced by older adults before coming to old age home (n=109)

Forms of abuse experienced by older adults	Frequency	Percentage
**Abuse total #**	**66**	**60.6**
Physical abuse	6	5.5
Caregiver neglect	38	34.9
Verbal abuse	38	34.9
Confinement	10	9.2
Financial abuse	3	2.8
Sexual abuse	8	7.3

#: Mean score (1.76$\pm$ 2.15) (among abused, minimum score for abuse 1, maximum score 9)
