# Supplementary material for: Abuse of older adults before moving to old age homes in Pokhara Lekhnath Metropolitan City, Nepal: A cross-sectional study
Source: PLoS One. 2021 May 7;16(5):e0250639. doi: 10.1371/journal.pone.0250639 (PMC8104417; doi:10.1371/journal.pone.0250639)
Supplement: S6 Table — (PDF) [file pone.0250639.s007.pdf]

Table 1: Factors associated with abuse of older adults transferred to old age homes (n=109)

Background characteristics	Crude Odds(95% CI)	Adjusted Odds(95%CI) ¤
**Age**		
Up to 70 years	2.508(1.141-5.516)[a]	2.318(0.951-5.650)
71 years and above	1	1
**Sex**		
Female	3.125(1.399-6.981)[b]	4.430(1.695-11.577)[b]
Male	1	1
**Education**		
Illiterate	0.481(0.182-1.268)	0.318(0.090-1.128)
Literate	1	1
**Marital Status**		
Unmarried/widowed/separated /divorced	0.460(0.205-1.035)	0.964(0.343-2.711)
Married	1	1
**Place of Residence**		
Municipality	1.476(0.642-3.399)	1.062(0.403-2.802)
VDC	1	
**Adequacy of annual income for 1 year**		
Inadequate	0.933(0.402-2.163)	0.734(0.268-2.007)
Adequate	1	1
**Before coming to the old age home, lived with**		
Other than family	0.474(0.215-1.047)	0.643(0.242-1.712)
With family	1	1
**Before coming to old age home**		
Did not have chronic disease	0.483(0.221-1.054)	0.539(0.224-1.297)
Had chronic disease	1	1
**Before coming to old age home**		
Independent for ADLS	12.154(0.457-10.141)	1.975(0.326-11.984)
Dependent on others for ADLS	1	1

a=$p<0.05$

b=$p<0.01$

¤ :The variables included in adjustment model were age, sex, education, marital status, place of residence, adequacy of annual income, living with status, chronic disease, and dependency for ADLs
